# Integrative Proteomic and Phosphoproteomic Analyses of Pattern- and Effector-Triggered Immunity in Tomato

**DOI:** 10.3389/fpls.2021.768693

**Published:** 2021-12-03

**Authors:** Juanjuan Yu, Juan M. Gonzalez, Zhiping Dong, Qianru Shan, Bowen Tan, Jin Koh, Tong Zhang, Ning Zhu, Craig Dufresne, Gregory B. Martin, Sixue Chen

**Affiliations:** ^1^Henan International Joint Laboratory of Agricultural Microbial Ecology and Technology, College of Life Sciences, Henan Normal University, Xinxiang, China; ^2^Department of Biology, Genetics Institute, Plant Molecular and Cellular Biology Program, University of Florida, Gainesville, FL, United States; ^3^Boyce Thompson Institute for Plant Research, Ithaca, NY, United States; ^4^Thermo Fisher Scientific Inc., West Palm Beach, FL, United States; ^5^Plant Pathology and Plant-Microbe Biology Section, School of Integrative Plant Science, Cornell University, Ithaca, NY, United States

**Keywords:** tomato, pattern-triggered immunity, effector-triggered immunity, proteomics, phosphoproteomics, *Pseudomonas syringae*

## Abstract

Plants have evolved a two-layered immune system consisting of pattern-triggered immunity (PTI) and effector-triggered immunity (ETI). PTI and ETI are functionally linked, but also have distinct characteristics. Unraveling how these immune systems coordinate plant responses against pathogens is crucial for understanding the regulatory mechanisms underlying plant defense. Here we report integrative proteomic and phosphoproteomic analyses of the tomato-*Pseudomonas syringae (Pst)* pathosystem with different *Pst* mutants that allow the dissection of PTI and ETI. A total of 225 proteins and 79 phosphopeptides differentially accumulated in tomato leaves during *Pst* infection. The abundances of many proteins and phosphoproteins changed during PTI or ETI, and some responses were triggered by both PTI and ETI. For most proteins, the ETI response was more robust than the PTI response. The patterns of protein abundance and phosphorylation changes revealed key regulators involved in Ca^2+^ signaling, mitogen-activated protein kinase cascades, reversible protein phosphorylation, reactive oxygen species (ROS) and redox homeostasis, transcription and protein turnover, transport and trafficking, cell wall remodeling, hormone biosynthesis and signaling, suggesting their common or specific roles in PTI and/or ETI. A NAC (NAM, ATAF, and CUC family) domain protein and lipid particle serine esterase, two PTI-specific genes identified from previous transcriptomic work, were not detected as differentially regulated at the protein level and were not induced by PTI. Based on integrative transcriptomics and proteomics data, as well as qRT-PCR analysis, several potential PTI and ETI-specific markers are proposed. These results provide insights into the regulatory mechanisms underlying PTI and ETI in the tomato-*Pst* pathosystem, and will promote future validation and application of the disease biomarkers in plant defense.

## Introduction

Plants are vulnerable to infection by a variety of microbial pathogens. During evolution, plants have developed two interlinked immune systems: pattern-triggered immunity (PTI) and effector-triggered immunity (ETI). Through cell surface-localized pattern recognition receptors (PRRs), plants can recognize conservative microbe- or pathogen-associated molecular patterns (MAMPs or PAMPs), leading to PTI (Bjornson and Zipfel, 2021). PTI responses include production of reactive oxygen species (ROS), activation of mitogen-activated protein kinase (MAPK) cascades, changes in the intracellular calcium concentration and transcriptional reprogramming of immunity-related genes, cell wall callose deposition, stomatal closure, and moderate inhibition of pathogen growth (Couto and Zipfel, 2016; Li et al., 2016, 2020). Through these responses, PTI provides an initial layer of defense against pathogens. Host-adapted pathogens such as *Pseudomonas syringae* DC3000 have evolved strategies to undermine PTI by delivering virulence factors (effectors) into plant cells (Gust et al., 2017). In turn, plants have evolved intracellular resistance proteins (mainly pathogen-specific nucleotide-binding leucine-rich repeat (NLR)-type immune receptors) capable of sensing specific effectors. This layer of defense, termed ETI, initiates an incompatible interaction characterized by induction of salicylic acid and systemic acquired resistance, defense gene transcription, as well as a hypersensitive response involving programmed cell death that limits pathogen spread (Cui et al., 2015; Li et al., 2020; Lu and Tsuda, 2021).

Recent discoveries of various immunogenic triggers and their receptors have revealed ambiguities in the distinction between PAMPs and effectors, as well as between PTI and ETI (Thomma et al., 2011; Ngou et al., 2021; Yuan et al., 2021a,b). PTI and ETI share some common immune responses, such as the generation of apoplastic ROS, activation of Ca^2+^ and MAPK cascades, production and secretion of antimicrobial compounds. Although the same or similar signaling components or enzymes are involved in PTI and ETI, the dynamics and strength of the activated responses are different. The activated immune responses during ETI are generally more prolonged and robust than those during PTI (Dodds and Rathjen, 2010; Tsuda and Katagiri, 2010; Thomma et al., 2011). However, it is currently unclear how PRRs and NLRs, localized to different cellular compartments, activate similar components to induce related defense outputs. Unraveling how PTI and ETI coordinate the immune responses is a grand challenge in plant defense biology.

*Pseudomonas syringae* pv. *tomato* (*Pst*), a hemibiotrophic bacterial pathogen, causes bacterial speck disease in different tissues of plants in the *Solanaceae* family, such as tomato (*Solanum lycopersicum*) (Jones et al., 1991). The tomato-*Pst* pathosystem has been extensively used as a model for studying the molecular mechanisms of plant-pathogen interactions (Oh and Martin, 2011; Velásquez and Martin, 2013; Zhang et al., 2020). Previous studies have reported that flagellin-derived MAMPs in *Pst* are the primary elicitors of PTI in tomato (Rosli et al., 2013). Flg22 and flgII-28, two MAMPs from the motility-associated flagellin protein encoded by the *fliC* gene, are recognized by the plant PRRs, FLAGELLIN SENSING 2 (FLS2) and FLAGELLIN SENSING 3 (FLS3), respectively (Gómez-Gómez and Boller, 2000; Hind et al., 2016). In addition, FLS2 and FLS3 associate with their co-receptor BRASSINOSTEROID INSENSITIVE1-ASSOCIATED RECEPTOR KINASE 1 (BAK1) to activate downstream immune signaling pathways (Sun et al., 2013; Hind et al., 2016). To overcome PTI, *Pst* DC3000 delivers a suite of 36 virulence-promoting effectors into the plant cells during the infection process (Wei et al., 2018). Two of the effectors, AvrPto and AvrPtoB act early in the tomato-*Pst* interaction by interfering with PRR functions and plant immune responses, thereby suppressing PTI and promoting bacterial virulence and multiplication (Martin, 2012). In response to *Pst* effectors, tomato evolved the Pto kinase which interacts with AvrPto/AvrPtoB, and works in concert with the NLR protein Prf to activate ETI (Martin et al., 1993; Salmeron et al., 1996; Martin, 2012). Although phosphorylation-mediated activation of FLS2-BAK1 and Pto-Prf has been well-studied, there are still many other unknown regulators to be explored during the PTI/ETI phosphorylation signaling pathway (Lei et al., 2020; Xue et al., 2020).

High-throughput quantitative transcriptomics and proteomics have increasingly been used in studying plant-pathogen interactions (Elmore et al., 2021). They help to characterize the changes in gene expression and protein levels during PTI and ETI in the tomato-*Pst* pathosystem, leading to discovery and functional characterization of some key MAMPs, PRRs, effectors, and NLRs (Parker et al., 2013; Rosli et al., 2013; Pombo et al., 2014). For example, Pombo et al. (2014) used treated Rio Grande tomato plants having a functional *Pto Prf* pathway (RG-PtoR) with a variety of *Pst* DC3000 mutants capable of eliciting of FLS2-mediated PTI or Pto/Prf-mediated ETI. A *Pst* DC3000 mutant lacking flagellin (Δ*fliC*) was used to inhibit FLS2-PTI while allowing ETI induced by AvrPto and AvrPtoB. A DC3000 double mutant defective in these two effectors (Δ*avrPto*Δ*avrPtoB*) was used to inhibit Pto/Prf-mediated ETI while eliciting FLS2-meidated PTI. A DC3000 triple mutant (Δ*avrPto* Δ*avrPtoB* Δ*fliC*) was used as a negative control where neither FLS2-PTI nor Pto/Prf-ETI could occur. This experimental design allowed the identification of several candidate genes using RNA-seq, including genes associated with flagellin-activated PTI (Δ*avrPto* Δ*avrPtoB* vs. Δ*avrPto* Δ*avrPtoB* Δ*fliC*) and Pto/Prf-mediated ETI (Δ*fliC* vs. Δ *avrPto* Δ*avrPtoB* Δ*fliC*). Notably, of the 64 protein kinase families present in tomato, 46 (72%) had at least one member induced in ETI, PTI or both. This highlights the importance of protein phosphorylation as a key post-translational modification regulating immune signal transduction. Previous phosphoproteomic studies have identified a large number of differentially phosphorylated proteins during PTI (Benschop et al., 2007; Nühse et al., 2007; Rayapuram et al., 2014; Mattei et al., 2016) and ETI (Kadota et al., 2019). However, all these studies were performed on model plant Arabidopsis, and the large-scale phosphoproteomics of PTI and ETI in the same study has not been reported.

In this study, we aimed to follow up the transcriptomic study by conducting proteomic and phosphoproteomic characterization of PTI and ETI responses using the same experimental design and tomato-*Pst* pathosystem (Pombo et al., 2014). Our results have improved understanding of the regulatory mechanisms of PTI and ETI at the protein level, which may inform strategies for improving crop disease resistance.

## Materials and Methods

### Plant Material and Bacterial Strains

Tomato Rio Grande-PtoR resistant plants (RG-PtoR) were grown at 76% humidity with a 16 h light/8 h dark cycle and a temperature of 23°C. *Nicotiana benthamiana* was kept in a growth chamber at 50% relative humidity with 16 h light/8 h dark cycle and a temperature of 22°C.

*Pst* DC3000 mutants (Δ*fliC*, Δ*avrPto* Δ*avrPtoB*, and Δ*avrPto* Δ*avrPtoB* Δ*fliC*) were grown on King’s Broth medium with ampicillin (100 μg/ml), kanamycin (50 μg/ml), and rifampicin (10 μg/ml) at 30°C (Pombo et al., 2014). The flagellin deficient strain (Δ*fliC*) was used to induce Pto/Prf ETI, and the double mutant of two effectors (Δ*avrPto*Δ*avrPtoB*) was used to elicit FLS2-mediated PTI. The triple mutant (Δ*avrPto*Δ*avrPtoB*Δ*fliC*) was a negative control where neither FLS2-PTI nor Pto/Prf-ETI occurred.

### Bacterial Infiltration and Induction of PTI and ETI

Four-week-old tomato leaves were vacuum infiltrated with a bacterial suspension of 5 × 10^6^ colony-forming-unit (cfu)/mL of different *Pst* DC3000 mutants described in the previous section. Leaf samples were collected 6 h after infection (hai), frozen in liquid N_2_, and stored at −80°C until processed. Four biological replicates were conducted.

### Protein Extraction and Quantitation

Proteins were extracted using a modified phenol extraction procedure (Hurkman and Tanaka, 1986). Briefly, for each sample, 2 g of leaf tissues were ground in liquid nitrogen. After adding 2 mL of Tris-buffered phenol and 2 mL of extraction buffer (0.1 M Tris–HCl at pH 8.0, 0.9 M sucrose, 0.4% β-mercaptoethanol, and 10 mM EDTA) containing 1 × protease and phosphatase inhibitor cocktails (Thermo Scientific Inc., Eugen, OR, United States), the samples were homogenized for 2 h. The homogenates were centrifuged at 5000 × *g* for 10 min at 4°C, and the phenol phase was added to five volumes of 0.1 M ammonium acetate in methanol and incubated overnight in a freezer. The protein precipitates were further washed with 0.1 M ammonium acetate in methanol twice, 80% acetone twice, and 100% acetone with centrifugation at 20,000 × *g* for 20 min at 4°C. Protein pellets were dissolved in a solubilization buffer (6 M Urea, 1 mM EDTA, 50 mM Tris–HCl at pH 8.5, 1% SDS, 10 mM DTT). The protein concentration was determined with an EZQ Protein Quantitation Kit (R-33200) according to the manufacturer’s instructions (Thermo Scientific Inc., Eugen, United States). A total of four independent biological replicates were carried out.

### Protein Digestion and TMT Labeling

A total of 500 μg proteins from each sample were reduced with 10 mM DTT for 1 h at 37°C, alkylated with iodoacetamide for 1 h at room temperature in the dark, and then digested with trypsin (Promega, Fitchburg, WI, United States) for 14 h at 37°C. After desalting using C18 solid phase extraction columns to remove the components that may interfere with the labeling (The Nest Group, Southborough, MA, United States), the peptides from three samples infected with different *Pst* DC3000 mutants were labeled with tandem mass tag (TMT) 6-plex tags (126 and 127, 128 and 129, and 130 and 131 for the leaf samples treated with *Pst* DC3000 Δ*fliC* mutant, Δ*avrPto*Δ*avrPtoB* mutant, and Δ*avrPto*Δ*avrPtoB* Δ*fliC* mutant, respectively) according to the manufacturer’s instructions (Thermo Scientific Inc., Eugen, United States). Labeled peptides were combined, and then desalted using C18 columns and lyophilized. Two independent TMT experiments were done to process four biological replicates of each sample type.

### Liquid Chromatography Fractionation and Phosphopeptide Enrichment

The peptide mixture was dissolved in 100 μL strong cation exchange (SCX) buffer (25% acetonitrile, 10 mM ammonium formate, and 0.1% formic acid, pH 2.8). Then it was fractionated on an Agilent HPLC 1260 with a SCX column (PolySULFOETHYL A, 100 × 2.1 mm, 5 μm, 300 Å), according to a previously described method (Yin et al., 2017). A total of 12 fractions were collected, desalted, and lyophilized. The fractionated peptides were dissolved in 30 μL of sample solvent (3% acetonitrile (ACN), 97% H_2_O, 0.1% acetic acid, and 0.01% trifluoroacetic acid (TFA)) and 30 μL of binding solution (80% ACN, 5% TFA), and then subjected to TiO_2_ + ZrO_2_ NuTips (equilibrated with binding buffer) (Glygen, Columbia, MD, United States) for phosphopeptide enrichment. The NuTip columns were washed with 80% ACN and 1% TFA, and the flow-through and washes were collected for the whole proteome analysis. Then, phosphopeptides were eluted from the column using 3% ammonium hydroxide. Phosphopeptides and the flow-through were quickly lyophilized for reverse phase liquid chromatography tandem mass spectrometry (LC-MS/MS).

### Reverse Phase LC-MS/MS

The peptide samples were separated using Easy-nLC 1000 (Thermo Scientific Inc., Germering, Germany) with a C18 reverse phase EASY-Spray nanoflow column (150 mm, 75-μm internal diameter, 2 μm, 100 Å). The peptides were eluted with a 120 min gradient with 95% solvent A (2% ACN, 0.1% formic acid (FA)) and 5% solvent B (0.1% FA, 98%ACN) to 15% solvent A and 85% solvent B at a flowrate of 300 nL/min at 30°C. MS/MS analysis was carried out on a Q-Exactive Orbitrap Plus ^TM^ mass spectrometer (Thermo Scientific Inc., Bremen, Germany). The MS1 scan was performed from 400–2000 m/z at a resolution of 70,000 with an automatic gain control (AGC) target of 1e6 and a maximum injection time (IT) of 100 ms. The top ten most intense ions were selected for MS2 scan at a resolution of 17,500, with a precursor isolation window of 1.3 m/z, a normalized collision energy of 35, the fixed first mass of 105, an AGC of 5e5, and a maximum IT of 55 ms. The underfill ratio was 1% and the charge exclusion was 1, 6–8, and >8.

### Database Searching and Quantitative Proteomic Analysis

The raw data files were analyzed for protein identification and quantification using the Proteome Discoverer 2.4 software (Thermo Scientific Inc., Bremen, Germany) with the Sequest HT algorithm. The spectra were searched against the ITAG 4.1 protein database (retrieved from the Sol Genomics Network, https://solgenomics.net/ftp//genomes/Solanum_lycopersicum/annotation/, 34,434 entries, downloaded on February 23, 2020). The precursor mass tolerance was set to 10 ppm, and the fragment mass tolerance was set to 0.02 Da. Dynamic modifications were allowed for phosphorylation on serine/threonine/tyrosine (+79.996 Da), TMT 6-plex reagents on lysine and peptide N-terminus (+229.163 Da), oxidation on methionine (+15.995 Da), and acetylation of protein N-termini (+42.011 Da). Carbamidomethylation (+57.021 Da) on cysteine residues were set as static modifications. The percolator had a false discovery rate of 0.05. For phosphoproteomic analysis, unique phosphopeptides with high confidence (99%) were selected for further analyses. The phosphorylation level was normalized against the whole proteome to determine the alteration of phosphorylation level rather than the change in protein abundance (Wu et al., 2011). For the total proteomic analysis, proteins quantified with high confidence and at least two peptides (including at least one unique peptide) were considered for further analyses. To determine differentially regulated (DR) proteins and phosphopeptides in a comparison set between two samples, a Student’s *t* test was performed and a cut-off of *p* < 0.05 was applied. Differentially regulated proteins/phosphopeptides with statistical significance between ETI-inhibited sample (Δ*avrPto*/*B*) and negative control (Δ*avrPto/B* Δ*fliC*) were considered as PTI-associated, and those between PTI-inhibited sample (Δ*fliC*) and negative control (Δ*avrPto/B* Δ*fliC*) were considered as ETI-associated.

### Bioinformatic Analysis of the DR Proteins/Phosphopeptides

Hierarchical clustering was performed based on log_2_ ratio of the differential abundance during PTI/ETI activation using the R-package “pheatmap.” Enrichment analyses of gene ontology (GO) biological processes and KEGG pathway, molecular function, and cellular component of DR proteins/phosphoproteins compared to the Arabidopsis reference gene background were performed using a Metascape online tool (Zhou et al., 2019). Terms with a *p*-value < 0.01, a minimum count of 3, and an enrichment factor >1.5 (the enrichment factor is the ratio between the observed counts and the counts expected by chance) are collected and grouped into clusters based on their membership similarities.

Protein-protein interaction (PPI) networks of the DR proteins/phosphoproteins were constructed using STRING tools with the medium confidence ≥ 0.4, which was then visualized using Cytoscape (3.7.1) software (Otasek et al., 2019). The Cytoscape MCODE (Multi-Contrast Delayed Enhancement) plug-in was used to search for clustered sub-networks, and the default parameters were as follows: degree cutoff = 2; node score cutoff = 0.2; K-core = 2; max depth = 100 (Bader and Hogue, 2003). To investigate amino acid frequencies around the phosphosites, the 12 surrounding amino acids (6 amino acids upstream and downstream of the site) were retrieved to generate a list of “phosphor-13-mers” using an OmicShare tool. Their motifs were extracted using the Motif-X algorithm in the MoMo tool (Version 5.0.5) (Cheng et al., 2019). Motif-X default settings of width = 13, occurrence = 20, and significance = 0.000001 were used.

Three-dimensional structural models of the DR phosphoproteins were generated on the basis of sequence alignments of the phosphoproteins (target) with the most similar protein (template) by using the SWISS-MODEL comparative protein modeling server^1^ (Biasini et al., 2014). The model was selected according to the QMEAN and GMQE scores, as well as high sequence identity (>30%). Functional domains were predicted using InterPro: the integrative protein signature database 13^2^. The three-dimensional structures and functional domains were visualized by Swiss-PdbViewer (version 3.7).

### Quantitative Real-Time (qRT-PCR) Analysis of Potential Markers for PTI and ETI

The sequences of candidate genes were retrieved from the ITAG 4.1 database (Sol Genomics Network). Specific primer pairs used in qRT-PCR were designed using NCBI Primer-Blast^3^ (Supplementary Table 1). Total RNA from four-week-old tomato leaves that were treated with different *Pst* mutants was isolated with an OminiPlant RNA Kit (Cwbiotech, Beijing, China), and then reverse-transcribed using HiScript^®^ II Q RT SuperMix for qPCR kit (+ gDNA wiper) (Vazyme Biotech, Nanjing, China) according to the manufacturer’s protocol. qRT-PCR was performed on a LightCycler^®^ 96 real-time PCR system (Roche, HongKong, China) using a SYBR Green Master Mix (Vazyme Biotech, Nanjing, China) according to the manufacturer’s instructions. Three biological and three technical replicated were conducted. The *actin* and *ubiquitin* genes were used as internal references.

### Construction of PTI-Specific Reporter and Assay for Reporter Activity After PTI Treatment

The promoter regions (∼1 kb upstream of gene start site) from NAC (NAM, ATAF and CUC family) domain protein (Solyc02g069960) and potential lipid particle serine esterase (SE) (Solyc04g077180) were amplified by nested PCR with primers (Supplementary Table 1), and fused to the binary vector pJM348 containing a *GUS A* intron. The recombinant plasmids *NACp*:*GUS*, *SEp*:*GUS*, *35S*:*GUS*, and *YFP* were introduced into *Agrobacterium tumefaciens* by electroporation.

Four-week-old *Nicotiana benthamiana* and tomato leaves were syringe-infiltrated with the agrobacteria in an infiltration buffer (10 mM MES pH 5.6, 10 mM MgCl_2_, 0.002% silwet and 0.1% acetosyringone) at OD_600_ of 0.5 (∼8.3 × 10^6^ cfu/mL). Each leaf was infiltrated with the four constructs in the agrobacteria: *YFP* (negative control), *35S:GUS* (positive control), *NACp*:*GUS* and *SEp*:*GUS*. After 48 h, each leaf was treated with the buffer (negative control) or a PTI-inducing treatment to create an overlapping pattern between the *Agrobacterium* transformation and the treatment. The PTI-inducing treatments were either 100 nM flg22 peptide or *Pseudomonas fluorescens* in the infiltration buffer with an OD_600_ of 0.5 (∼8.3 × 10^6^ cfu/mL). The leaf tissue was harvested at 6, 12, and 16 hai. The reporter activity was assayed by vacuum infiltration of the leaves with a GUS staining solution (1 mM X-Gluc, 100 mM Sodium Phosphate pH 7, 0.1% Triton X-100, 10 mM EDTA). The leaves were placed in ethanol and acetic acid (3:1 by volume) until they became transparent.

## Results

### Changes of Proteome and Phosphoproteome in Tomato Leaves Upon PTI and ETI Activation

To profile protein expression and phosphorylation events during PTI and ETI activation, we applied a multiplexed proteomics approach to quantify the proteome and phosphoproteome of tomato leaves infiltrated by different *Pst* DC3000 mutants, which allow for the dissection of the specific plant immune responses. We collected leaf tissues at 6 h after inoculation to assess the changes of host protein expression and phosphorylation after *Pst*-mediated translocation of AvrPto and AvrPtoB into the plant cell, which occurs about 3 h after syringe infiltration of *Pst* (Pombo et al., 2014). Another batch of similarly inoculated plants were maintained in the same conditions to observe disease symptoms. It is known that RG-PtoR plants infiltrated with *Pst* DC3000 Δ*fliC* show no disease symptoms, but develop speck disease when infiltrated with a *Pst* strain lacking effectors AvrPto and AvrPtoB (Δ*avrPto*/*B* and Δ*avrPto*/*B* Δ*fliC* strains). Especially severe disease is observed in the triple mutant (Pombo et al., 2014).

**FIGURE 1:**
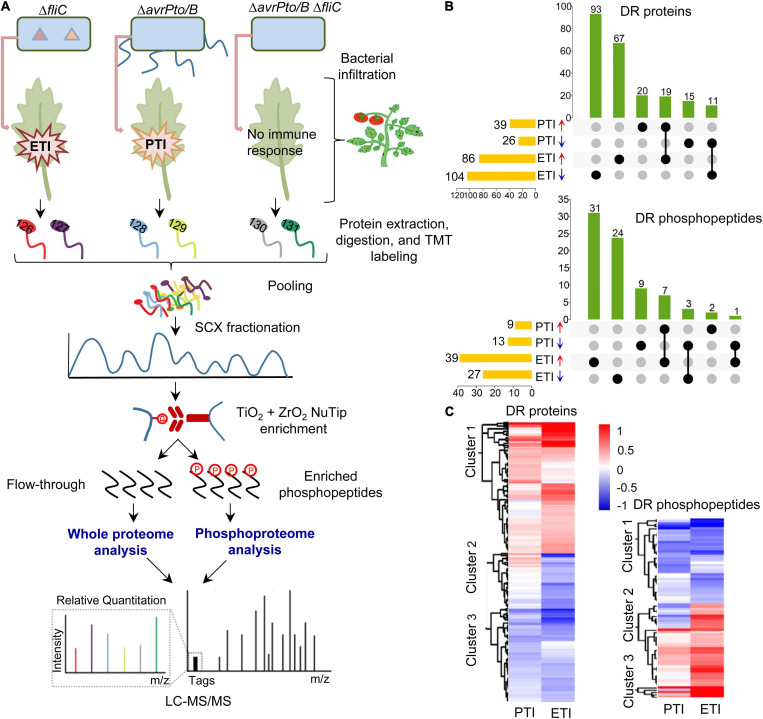
Overview of the proteomics and phosphoproteomics workflow and summary of the tomato PTI and ETI results. **(A)** Workflow for the proteomic analysis. Four-week-old tomato leaves were vacuum infiltrated with different *Pst* DC3000 mutants. Proteins were extracted from leaf samples, digested by trypsin, and labeled with TMT reagents. After fractionation by strong cation exchange (SCX) chromatography, the phosphopeptides were enriched by TiO_2_ + ZrO_2_ NuTips, the flow-through and phosphopeptides were submitted to reverse phase liquid chromatography tandem mass spectrometry (LC-MS/MS), and analyzed with Proteome Discoverer 2.4 software and bioinformatic tools; **(B)** Upset plot displaying the overlaps of differentially regulated (DR) proteins and phosphopeptides during PTI and ETI. Each column represents shared DR proteins/phosphopeptides among the comparisons (dots connected by lines below the X axis), with the comparisons on the left and the total number of DR proteins/phosphopeptides. The number of DR proteins/phosphopeptides in each set is shown on the top of the column; and **(C)** Hierarchical clustering analysis of DR proteins and phosphopeptides. The two columns represent different treatments of PTI and ETI. The rows represent individual proteins/phosphopeptides. The detailed information is listed in Supplementary Tables 4, 5. The increased or decreased proteins/phosphopeptides are indicated in red or blue, respectively.

As depicted in Figure 1A, proteins from the inoculated leaves were extracted, digested, and labeled with isobaric TMT tags, followed by SCX fractionation and TiO_2_/ZrO_2_-based phosphopeptide enrichment. The flow-through and enriched phosphopeptides were subjected to LC-MS/MS for whole proteome and phosphoproteome profiling, respectively. Thus, it allowed us to determine both protein abundance and phosphorylation changes associated with flagellin-activated PTI (Δ*avrPto*/*B* vs. Δ*avrPto/B* Δ*fliC*) and Pto/Prf-mediated ETI (Δ*fliC* vs. Δ*avrPto/B* Δ*fliC*). In total, 3097 proteins and 602 phosphopeptides were identified at 1% FDR. Among them, 225 proteins were differentially regulated (DR) during PTI/ETI (Supplementary Table 2) at the protein abundance level with 35 PTI-specific (20 increased and 15 decreased), 160 ETI-specific (67 increased and 93 decreased), and 30 responsive for both PTI and ETI (19 increased and 11 decreased) (Figure 1B).

Analysis of the phosphoproteome revealed 79 phosphopeptides that show significant changes during pathogen infection (Supplementary Table 3). The change in phosphorylation was further compared to that of protein abundance to accurately reflect the phosphorylation alteration. This is because the apparent phosphorylation change could be attributed to the changes at the protein abundance, as evidenced by a similar change at both levels for meloidogyne-induced giant cell protein (MI-GCP) following ETI treatment (0.72-fold and 0.79-fold change for Ser-525 and Ser-627 at the phosphorylation level and 0.79-fold at the protein level). After excluding such cases, 77 significantly changed phosphopeptides remained with 11 PTI-specific (including 2 increased and 9 decreased), 55 ETI-specific (31 increased and 24 decreased), and 11 responsive for both PTI and ETI, and among which only one phosphopeptide showed the opposite trend (Figure 1B). Thus, ETI triggered a more extensive reprogramming of both the proteome and phosphoproteome than PTI.

To explore the molecular patterns upon PTI and ETI activation, hierarchical cluster analysis was performed with the DR proteins/phosphopeptides based on their differential abundances (Supplementary Tables 4,5). In most cases, the PTI-associated proteins/phosphopeptides tend to change in the same direction, but the changes were much stronger in the ETI response (Figure 1C). These results support that PTI and ETI share signaling network machinery, and PTI response was transient and easily undermined by the pathogen, whereas the ETI response was more robust.

### Proteome Reprogramming by PTI and ETI Activation of Multiple Functional Modules

**FIGURE 2:**
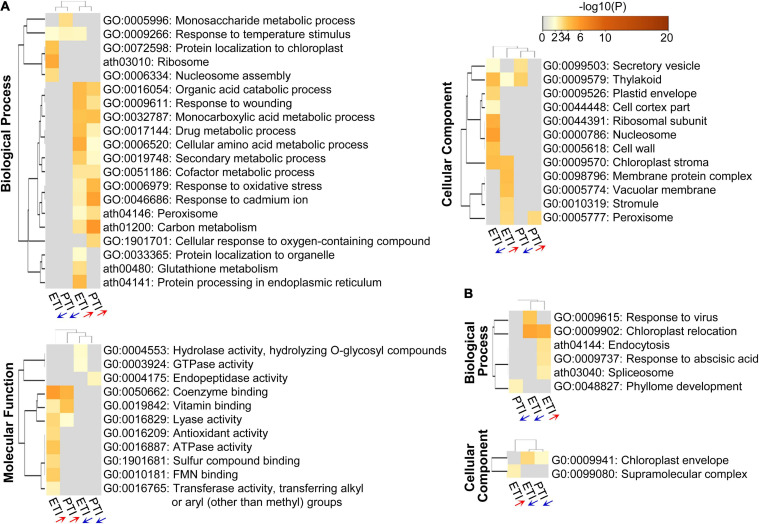
Heatmap of GO enriched terms across the input protein lists, colored by *p*-values. **(A)** DR proteins; and **(B)** DR phosphoproteins. The red or blue arrows below the columns indicate the lists containing increased or decreased proteins/phosphoproteins, respectively.

To gain further insight into the function and mechanisms of ETI and PTI, GO enrichment and KEGG pathway analyses were performed (Figure 2). Both ETI and PTI-induced proteins were predominantly enriched in the metabolic processes (including organic acid, amino acid, secondary metabolite, cofactor, and carbon metabolism), and responses to stresses (including responses to wounding, oxidative stress, and cadmium), while ETI-decreased proteins were significantly enriched in the assembly of ribosome and nucleosome (Figure 2A). Analysis of molecular function revealed that both ETI- and PTI-induced proteins were primarily enriched in coenzyme binding, vitamin binding, and lyase activity, while ETI-induced proteins are specifically enriched in the binding of sulfur compound and flavin mononucleotide, activities of antioxidant, ATPase, and transferase. At the cellular component level, the ETI-induced proteins and ETI-decreased proteins had diverse subcellular localizations. These results reflect the shared machinery and diversity in the biological functions of ETI and PTI.

**FIGURE 3:**
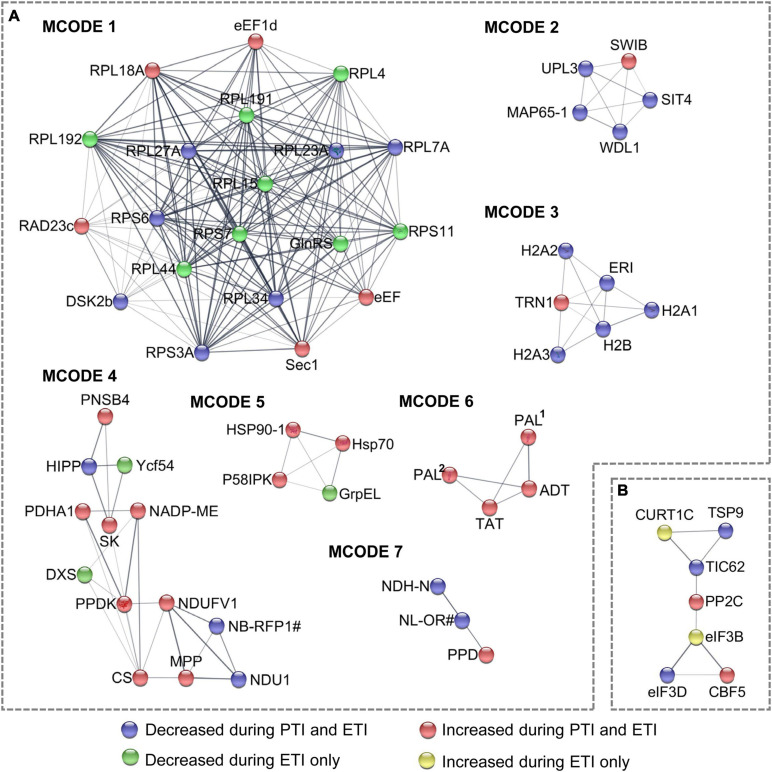
Subnet module analysis of the protein-protein interaction (PPI) network. **(A)** DR proteins; and **(B)** DR phosphoproteins. Different colors of the nodes correspond to the Clusters in Figure 1C, which represent different change patterns of the DR proteins and phosphoproteins, as shown at the bottom of the figure. refer to Supplementary Tables 2, 3 for abbreviations.

To model the dynamics of the proteome changes upon PTI/ETI activation, the DR proteins were superimposed onto the protein-protein interaction (PPI) network to identify functional modules. A network containing 160 nodes and 460 edges was constructed (Supplementary Figure 1A), and seven modules within which the protein members were highly inter-connected (Figure 3A). These modules were annotated to multiple biological processes (Supplementary Table 6). For example, the largest module (MCODE1) consisted of ribosome proteins, with majority decreased during both PTI and ETI or during ETI only. MCODE2 and MCODE3 was made up of five proteins and six proteins, respectively, and most of them were decreased during both PTI and ETI. The proteins in MCODE3 were mainly enriched in the chromatin organization. Additionally, proteins in MCODE4 were mainly enriched in the pyruvate metabolic process and the electron transport chain, and proteins in MCODE5 were enriched in protein folding. Interesting, MCODE6 consisted of four proteins, which were related to phenylalanine metabolic process and all increased during PTI and ETI.

### PTI and ETI Signaling Networks Revealed by Phosphoproteomics

Similar pathway analysis on phosphoproteins revealed that phosphoproteins increased by ETI are enriched in chloroplast relocation, endocytosis, response to abscisic acid, and spliceosome; while these decreased by ETI were enriched in chloroplast relocation and response to viruses (Figure 2B). For cellular component, phosphoproteins increased by ETI were enriched in supramolecular complex, while these decreased by PTI or ETI were enriched in chloroplast envelope. Superimposing these proteins onto the PPI network identified 31 nodes and 48 edges (Supplementary Figure 1B) with one functional subnet module selected (Figure 3B and Supplementary Table 6).

**FIGURE 4:**
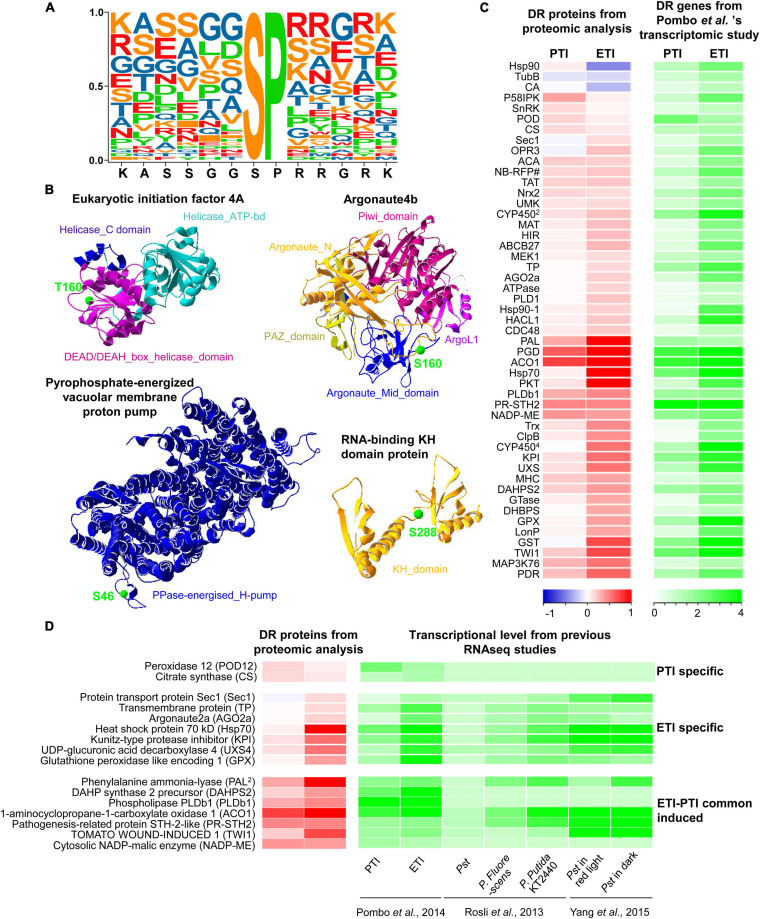
Extracted motifs and predicted 3D structures of DR phosphoproteins, as well as integrative analysis of proteome and transcriptome. **(A)** A motif extracted from 41 DR phosphosites. **(B)** 3D structures of four DR phosphoproteins. More detailed information can be found in Supplementary Table 8. **(C)** Heatmap of DR proteins with relative changes at the protein level and transcript level from Pombo et al. ’s transcriptomic study. The two columns represent different treatments of PTI and ETI. The rows represent individual proteins. The detailed information is listed in Supplementary Table 9. The increased or decreased proteins are indicated in red or blue, respectively, and the increased gene transcripts are shown in green. The log_2_ ratios of fold-change levels of proteins or transcripts are indicated at the bottom. **(D)** Heatmap of DR proteins with conserved changes at the protein level and transcript level from multiple RNA-seq studies. The increased or decreased proteins are indicated in red or blue, respectively, and the increased gene transcripts are shown in green.

Further motif enrichment analysis of the 83 phosphosites revealed that 41 contain a Ser-Pro (SP) motif (Figure 4A and Supplementary Table 7), a typically substrate of MAPKs and thus highlighting the important roles of MAPK signaling in plant immune responses (Pedley and Martin, 2004, Pedley and Martin, 2005; Muñoz et al., 2018). In addition, all the differentially regulated phosphoproteins were subjected to SWISS-MODEL database for building the 3D structure homology model to better understand the structure-function relationship. A total of 46 statistically acceptable homology models were built. Among them, the phosphorylation sites of only four phosphoproteins were located within the 3D structure models (Figure 4B and Supplementary Table 8). Remarkably, the phosphorylation sites of these proteins are localized in the functional domain, where they form loops or random coils in close proximity with the active sites. For instance, the phosphosite S160 in protein argonaute4b was localized in the random coil and the Argonaute_Mid_domain. These data provide new candidates for functional characterization of the phosphoproteins and phosphosites in the PTI and ETI signaling processes.

### Integration of Transcriptomic and Proteomic Data

To identify genes that are regulated at both protein and transcription levels, the proteome data were integrated with available RNA-seq data that used the same materials and inoculation methods (Pombo et al., 2014; Figure 4C, and Supplementary Table 9). In line with the proteomics data, we found increases in gene expression for 49 DR proteins including 17 PTI- and 41 ETI-increased proteins. Interestingly, we found that the protein abundances associated with three tomato ETI specific-induced genes (heat shock protein 90, tubulin beta chain and carbonic anhydrase) were actually reduced during ETI (Figure 4C). Additionally, to understand the conserved defense mechanisms in tomato, the proteomic data were also correlated with several other RNAseq datasets. We have found a number of proteins that were also induced in resistant (RG-PtoR) or susceptible (RG-prf3) tomato against a variety of *Pseudomonas syringae* pv. tomato mutants capable of eliciting of PTI or ETI, as well as two other members of the genus *Pseudomonas* (*P. fluorescens* and *P. putida* KT2240) (Rosli et al., 2013; Pombo et al., 2014; Yang et al., 2015) (Supplementary Table 10). These integrative analyses provide valuable information for exploring reliable marker for PTI or ETI, such as peroxidase 12 (POD12) and citrate synthase for PTI, as well as Hsp70, kunitz-type protease inhibitor (KPI), UDP-glucuronic acid decarboxylase 4 (UXS4), glutathione peroxidase like encoding 1, etc (Figure 4D). Moreover, a new set of proteins were also identified by our study by comparing with these RNAseq data (Supplementary Table 11). These newly identified proteins that function in tomato-*Pst* pathosystem highlight involvement of some unknown mechanisms, which may be characterized in future studies, such as PTI-induced UDP-glucuronic acid decarboxylase 1 and 2-oxoglutarate-dependent dioxygenase 2, as well as ETI-induced stolen tip protein TUB8-like isoform 1, transcriptional coactivator/pterin dehydratase, 2-oxoglutarate (2OG) and Fe (II)-dependent oxygenase, NADPH-cytochrome P450 reductase (CPR) (Table 1).

**TABLE 1:** Putative newly identified PTI-specific, ETI-specific, as well as common ETI and PTI-induced proteins.

Accession No.	Protein name	PTI		ETI	
		Ratio	*p* value	Ratio	*p* value
**Newly identified PTI-specific proteins**					
Solyc10g085920.3.1	UDP-glucuronic acid decarboxylase 1 (UXS1)	**1.223**	0.032	1.039	0.507
Solyc07g043420.3.1	2-oxoglutarate-dependent dioxygenase 2 (2OGDD2)	**1.208**	0.033	0.955	0.463
**Newly identified ETI-specific proteins**					
Solyc11g012320.3.1	Induced stolen tip protein TUB8-like isoform 1 (TUB8)	1.033	0.933	**1.636**	0.01
Solyc03g094175.1.1	Transcriptional coactivator/pterin dehydratase (PCDH)	1.148	0.343	**1.62**	0.009
Solyc09g089720.4.1	2-oxoglutarate (2OG) and Fe(II)-dependent oxygenase (2OGX)	1.087	0.015	**1.344**	0
Solyc07g019460.3.1	NADPH-cytochrome P450 reductase (CPR)	1.132	0.086	**1.236**	0.007
**Newly identified PTI-ETI-induced proteins**					
Solyc07g049645.1.1	Benzyl alcohol O-benzoyltransferase (BEBT)	**1.319**	0.031	**2.293**	0.008
Solyc05g013850.2.1	Sieve element occlusion b (SEOB)	**1.271**	0.006	**1.419**	0.004

*Bold numbers indicate significant changes in protein abundance.*

### Expression of Potential Markers for PTI and ETI

**FIGURE 5:**
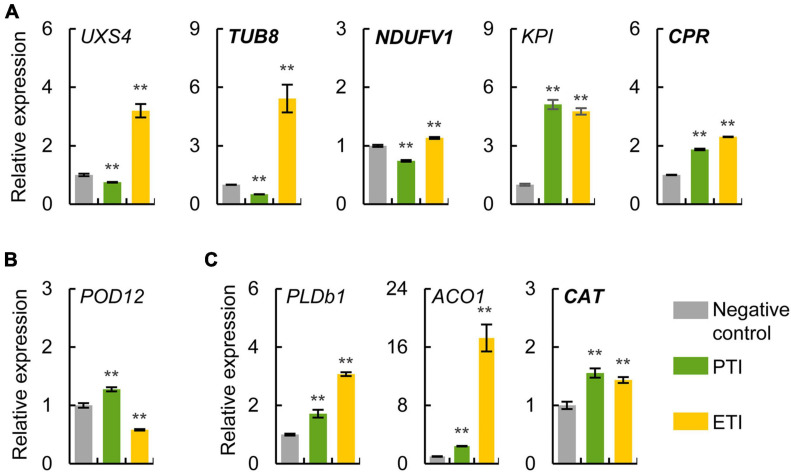
Expression analysis of potential markers for PTI and ETI using qRT-PCR. Relative mRNA expression levels of ETI-specific **(A)**, PTI-specific **(B)**, and common induced **(C)** proteins were determined in four-week-old tomato leaves treated with different *Pst* mutants to induce ETI or PTI. Normal italic and bold italic names indicate conserved defense-related proteins and newly identified proteins, respectively. The *actin* gene was used as an internal reference. The values were presented as mean ± SD (*n* = 3). Significant differences between negative control and treatment are marked with asterisks (**P < 0.01, Student’s *t* test). The abbreviations of the genes refer to their homologous proteins in Figure 4D and Supplementary Table 11.

To evaluate the expression patterns of potential markers for PTI and ETI, some genes encoding the conserved defense-related proteins and newly identified proteins were selected for qRT-PCR analysis. The gene expression patterns were determined in four-week-old tomato leaves infiltrated with different *Pst* DC3000 mutants to induce PTI or ETI. The expression patterns of these genes were similar based on different internal reference genes (*actin* and *ubiquitin*), indicating the reliability of the qRT-PCR results (Figure 5 and Supplementary Figure 2). We confirmed the mRNA expression levels of *UXS4*, *TUB8* and *NADH dehydrogenase [ubiquinone] flavoprotein 1* (*NDUFV1*) were ETI-specific induced, *POD12* was PTI-specific induced, as well as *phospholipase Db1* (*PLDb1*), *1-aminocyclopropane-1-carboxylate oxidase 1* (*ACO1*), and *catalase* (*CAT*) were common induced during both ETI and PTI, as found from the proteomic data (Figure 4D and Table 1, and Supplementary Table 11). However, ETI-induced KPI and CPR revealed by the proteomic analysis were also found to be up-regulated during PTI using qRT-PCR. These results implied that both transcriptional and translational regulations were involved in the plant immune responses.

### Conserved Differentially Phosphorylated Sites in Response to PTI and/or ETI Activation

In order to discover the conserved phosphorylation-mediated defense mechanisms in plants, we sought to query whether the corresponding sites of homologous proteins in Arabidopsis responded to PTI (Benschop et al., 2007; Nühse et al., 2007; Rayapuram et al., 2014; Mattei et al., 2016), ETI (Kadota et al., 2019), or *Pst* (Pang et al., 2020). Six of 83 PTI and/or ETI-regulated phosphorylation sites in tomato were found to be homologous to previously identified PTI/ETI-regulated phosphorylation sites in Arabidopsis (Table 2). Specifically, Ser-333 from PLASTID MOVEMENT IMPAIRED 1 was both ETI-decreased in tomato and Arabidopsis, and Ser-72 from molybdenum cofactor sulfurase was PTI-induced in tomato and ETI-induced Arabidopsis. Moreover, the ETI-decreased phosphorylation sites in tomato were found to be also decreased in Arabidopsis in response to *Pst*, including eukaryotic initiation factor 4A, ARF GAP-like zinc finger-containing protein ZIGA4, and chloroplastic protein TIC62. These results suggest the conserved mechanisms of these phosphorylation sites in plant immune responses.

**TABLE 2:** Conserved differentially phosphorylated sites in tomato and Arabidopsis in response to PTI and/or ETI activation.

	Tomato					Arabidopsis			
Protein name	PTI			ETI		Phosphorylation			
	Phosphorylation		Protein	Phosphorylation	Protein	ID	Site	Ratio	Treatment
PLASTID MOVEMENT IMPAIRED.1	S333	0.933	0.921	***0.905***	0.946	AT1G42550	S328	0.599 ^(1)^	ETI
Molybdenum cofactor sulfurase	S72	**1.113**	-	1.150	-	AT4G37100	S78	6.555 ^(1)^	ETI
Eukaryotic initiation factor 4A	T146	0.858	-	***0.793***	-	AT3G13920	T146	−2.000 ^(2)^	*Pst*
ARF GAP-like zinc finger-containing protein ZIGA4	S195	0.952	-	***0.838***	-	AT1G08680	S196	−2.000 ^(2)^	*Pst*
Protein TIC 62	T432	0.947	0.953	***0.828***	0.925	AT3G46780	T451	−2.000 ^(2)^	*Pst*
Late embryogenesis abundant protein	S29	0.814	-	***0.826***	-	AT1G45688	S30	1.500 ^(3)^	PTI

*Bold straight and bold italic numbers indicate increased and decreased phosphorylation levels, respectively.*

*^(1)^ The phosphorylation ratio upon ETI-inducing after 3 h of Dex treatment (Kadota et al., 2019).*

*^(2)^ The phosphorylation ratio at 60 min after *Pst* treatment (Pang et al., 2020).*

*^(3)^ The phosphorylation ratio at 10 min after flg22 treatment (Benschop et al., 2007).*

### Promoter Activities of Two PTI-Specific Genes Identified From Transcriptomics

NAC (Solyc02g069960) and SE (Solyc04g077180) were identified as PTI-specific markers for tomato-*Pst* interaction as they were induced specifically by PTI within 6 to 12 hai based on transcriptomics (Pombo et al., 2014). Our proteomics data, however, showed no differential protein expression following *Pst* infection suggesting that their associated proteins are likely not suitable as PTI markers.

**FIGURE 6:**
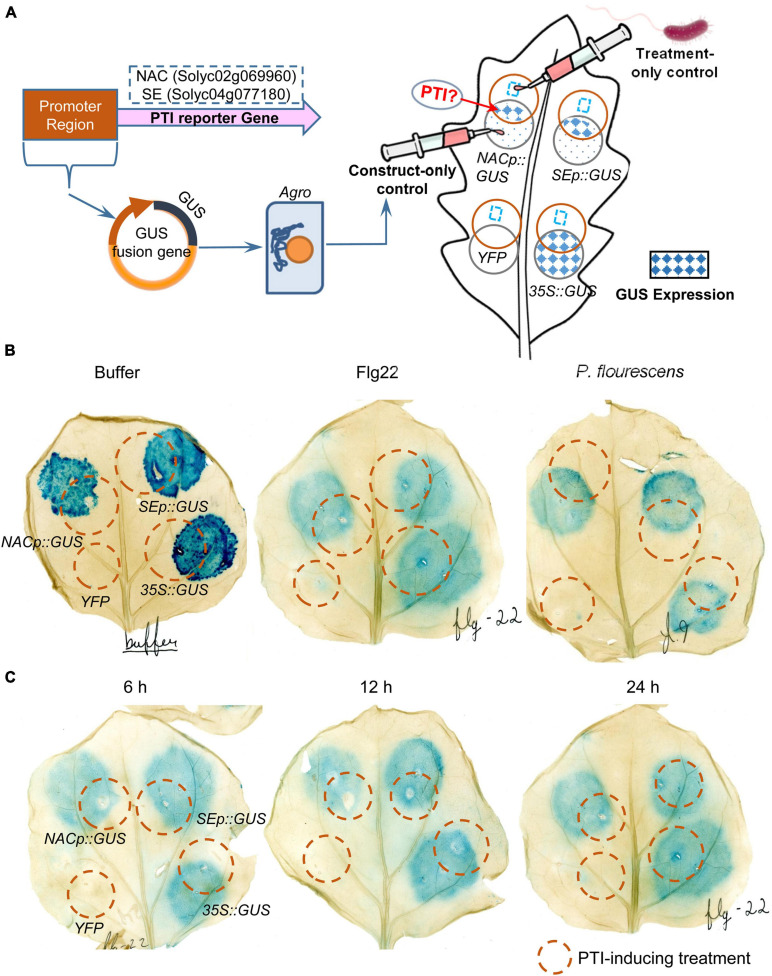
Transient expression assays of *NAC* and *SE* genes in *N. benthamiana* leaves in response to different PTI-inducing treatments at different time-points. **(A)** Schematic workflow of the assay and interpretation of the “overlapping circle” pattern permitted for a qualitative analysis of the GUS histochemical stain. Each leaf was infiltrated with four agrobacterium constructs: *YFP* (negative control), *35S*:*GUS* (positive control), *NACp*:*GUS* and *SEp*:*GUS*; **(B)** *N. benthamiana* leaves treated with negative control buffer, flg22, and *P. fluorescens* for 12 h and then GUS staining for 16 h; and **(C)** *N. benthamiana* leaves treated with flg22 for 6, 12, and 24 h and then GUS staining for 16 h.

To investigate the possible roles of NAC and SE in PTI, promoter-GUS fusion constructs using the promoter regions from NAC and SE were constructed and transiently expressed in *N. benthamiana* leaves. The “overlapping circle” pattern assay was used to quantify GUS expression and test the functionality and specificity upon PTI treatment (Figure 6A). After GUS staining, circular blue stains were observed with *35S*:*GUS*, *NACp*:*GUS*, and *SEp*:*GUS*, but not in the YFP negative control, suggesting functional and inducible *GUS* gene expression. However, no overlapping pattern (i.e., darker blue spots in the overlapping region after PTI activation) was observed in the leaves treated with negative control buffer, flg22, or *P. fluorescens* for 12 h (Figure 6B). Shortening the treatment time to 6 h or extending it to 24 h did not impact GUS expression, indicating that the *GUS* expression is independent of the PTI induction time (Figure 6C). To explore the possibility that over-staining might override possible differences in expression profiles between the treatments, the 12 hai treated leaf tissue was incubated in the X-Gluc staining buffer for variable time periods. The data showed that the 6 h timepoint was consistently lighter than the 11 h and 16 h timepoints regardless of the treatments (Supplementary Figure 3A). The results thus rule out the contribution from potential staining saturation. Important, the GUS expression read-out showed that there was no observable overlapping pattern and no difference in expression profiles between the different treatments, consistent with the previous assay results in Figure 6. The “overlapping circle” pattern assay was also carried out in the same way in tomato (RG-PtoR) (Supplementary Figure 3B). The resulting expression profiles were similar to those described above in the *N. benthamiana* assay, except that the *SEp*:*GUS* failed to yield a blue histochemical stain. These results thus indicate that NAC and SE are not PTI-specific and may not be appropriate as PTI-markers.

## Discussion

Although some key components and molecular processes of PTI and ETI have been studied for decades (Dodds and Rathjen, 2010; Zhang et al., 2013), the complete set of molecular components and interplay between PTI and ETI are not fully understood. Through transcriptomics, the genes specifically induced during PTI/ETI were identified in tomato leaves with a series of *Pst* DC3000 mutants capable of eliciting of PTI or ETI (Pombo et al., 2014). The study revealed potential involvement of genes in 46 protein kinase families in PTI/ETI, highlighting the importance of protein phosphorylation in the *Pst*-tomato interaction. To discover the proteins and phosphorylation events in the *Pst*-tomato interaction, we used the same tomato leaf samples and infiltrated with the same *Pst* DC3000 mutants as in the transcriptomics work (Pombo et al., 2014) for proteomics and phosphoproteomics in this study.

### Overlapping and Diverse Immune Responses Triggered by PTI and/or ETI

Our proteomic analyses revealed a larger number of ETI-associated changes than PTI-associated changes at both protein abundance and phosphorylation levels (Figures 1B,C). This is consistent with the transcriptomic results, where a high percentage of ETI-induced genes were not altered during PTI (Pombo et al., 2014). In addition, our results showed that PTI-associated proteins/phosphopeptides tend to change in the same direction and at a larger scale during the ETI response (Figure 1C). This is also consistent with the notion that PTI is an indispensable component of ETI during *Pst* infection (Yuan et al., 2021a). GO enrichment revealed that PTI- and ETI-responsive proteins shared some biological processes. For example, both ETI and PTI-induced proteins were enriched in several metabolic processes (including organic acid, monocarboxylic acid, amino acid, secondary metabolite, cofactor, and carbon metabolism), and stress response signaling (including responses to wounding, oxidative stress, and cadmium) (Figure 2A). These results support previous reports that PTI and ETI gene expression signatures are largely similar, but they vary in magnitude. PTI response was transient and easily suppressed by the pathogen, whereas the ETI response was more robust and prolonged (Dodds and Rathjen, 2010; Tsuda and Katagiri, 2010; Thomma et al., 2011; Xin and He, 2013). Here we identified a common set of differential proteins/phosphoproteins during PTI and/or ETI, which are involved in MAPK cascades, activation of Ca^2+^, and production/secretion of antimicrobial compounds.

MAPK cascades have been implicated in both PTI and ETI (Pitzschke et al., 2009). In this study, 49% of the DR phosphosites belong to MAPK phosphorylation motifs, suggesting these phosphoproteins are likely to be MAPK cascade components (Figure 4A). MPK3 was significantly induced during both PTI and ETI, and MAPK kinase 1 (MKK1) was increased during ETI (Supplementary Table 2). This result is consistent with a previous report that *At*MPK3 was activated during both PTI and ETI, and it was essential for the robust ETI (Tsuda et al., 2013). The much higher fold changes in these MAPK pathway components during ETI than PTI (Figure 1C) corroborate with the previous observation of long-lasting MAPK activation during ETI compared to PTI (Tsuda et al., 2013). *At*MKK1 was found to be required for the activation of flg22-induced MPK4, a key component of PTI pathway (Mészáros et al., 2006; Suarez-Rodriguez et al., 2007). The induced MKK1 upon ETI in this study suggesting MKK1 may also play a crucial role during ETI of the *Pst*-tomato interaction.

Activation of PRR signaling leads to a rapid and drastic increase of cytosolic Ca^2+^ concentration, which is important for subsequent immune responses during both PTI and ETI activation (Yu et al., 2017). Here we found that a calcium-transporting ATPase (ACA) (homolog to *At*ACA11) and calcium-dependent protein kinase 32-like (CPK32) were induced at the protein and transcript levels during both PTI and ETI (Figure 4C). ACA8 and its homolog ACA10 associate with FLS2 and are required for flg22-induced PTI (Frey et al., 2012). The tonoplast-localized ACA11 may contribute to the elevation of cytosolic Ca^2+^ concentration through Ca^2+^ mobilization from the vacuole stores (Lee et al., 2007). *Sl*CPK32 were also induced at 5 days after *Stemphylium lycopersici* inoculation in the *Sm* tomato cultivar (Yang et al., 2017). However, *At*CPK32 was previously suspected to act as a negative regulator in Plant Elicitor Peptide 1-induced early defense signaling, including ROS production, activation of MAPK cascade, and defense gene expression (Wang, 2018). The different mechanisms in Arabidopsis and tomato of CPK32-regulated immune signaling pathways remain to be elucidated. Additionally, two proteins related to ETI signaling were found to be increased by ETI in this study (Supplementary Table 2). A hypersensitive-induced response protein (HIR) was induced both in protein and transcript abundances during ETI only (Figure 4C). This is consistent with a previous study that *At*HIR1 acts as a potential RESISTANCE TO PSEUDOMONAS SYRINGAE 2 (RPS2) component in RPS2-mediated ETI (Qi et al., 2011). Furthermore, a SIT4 phosphatase family protein, known as the catalytic subunit of a protein phosphatase 2A (PP2A)-like protein phosphatase, was also increased in ETI. PP2A has been previously related to ETI in tobacco. It negatively regulates phosphorylation levels of the plasma membrane receptor kinase BAK1, and inhibits PTI responses (He et al., 2004; Segonzac et al., 2014). Moreover, protein phosphatase 2C-19 (PP2C19) and the blue light receptor phototropin-2 (Phot2) identified as plant PP2A substrates showed enhanced phosphorylation at Ser-55 and Ser-358, respectively, in ETI. PP2Cs negatively regulate pattern recognition receptor elongation factor Tu receptor and Xanthomonas resistance 21, a receptor mediating resistance to *Xanthomonas oryzae* pv. *Oryzae* (*Xoo*) in rice (Park et al., 2008; Couto et al., 2016). Previous studies revealed that Phot2 could interact with RPS2, and was required for hypersensitive response to turnip crinkle virus (Jeong et al., 2010). How PP2C19 and Phot2 are regulated by phosphorylation and PP2A upon *Pst* infection warrants future investigation.

We also identified several known defense-related proteins in this study. One pathogenesis-related (PR)-10 family protein salt tolerance homolog 2 (STH-2) was significantly induced at both transcription and protein levels during both PTI and ETI (Figure 4C). It was reported to be increased in tomato leaves in response to *Phytophthora infestans* (Fan et al., 2021). Interestingly, phosphorylation of a conserved immunity regulator RPM1-interacting protein 4 (RIN4) (Zhao et al., 2021) was significantly increased (e.g., Ser-144) during both PTI and ETI (Supplementary Table 3). Phosphorylation on multiple residues of RIN4 has been implicated in the regulation of plant immunity (Lee et al., 2015). For example, phosphorylation of *At*RIN4 Thr-166 plays contrasting roles in suppressing PTI and activating ETI (Toruño et al., 2019). The increased phosphorylation of *Sl*RIN4 at Ser-144 during both PTI and ETI implies potential new functions of this Ser residue in the tomato-*Pst* interaction.

### Perturbation of Redox Homeostasis Upon PTI and/or ETI Activation

While PTI induces a rapid and transient ROS burst, ETI is usually associated with a much stronger and sustained ROS burst (Yuan et al., 2021b). Respiratory Burst Oxidase Homolog (RBOH) is a major enzyme that mediates the production of ROS during PTI and ETI (Kadota et al., 2019). However, RBOHB (RBOHD in Arabidopsis) was found increased only during ETI (Supplementary Table 2). This is consistent with the results from two recent studies, i.e., PRR signaling requires for maximal phosphorylation of RBOHD during ETI, whereas NLR signaling upregulates the levels of RBOHD (Ngou et al., 2021; Yuan et al., 2021a). *Sl*RBOHD was also found to be stimulated at the late stage of *Cladosporium fulvum* infection in resistant line of Cf-12 tomato (Xue et al., 2017). Therefore, the increase of *Sl*RBOH protein level may help enable robust ROS production during ETI. In addition to RBOH-mediated ROS, ROS can also be generated by class III POD (Daudi et al., 2012). *Sl*POD12, a member of the class III apoplastic POD, displayed increased abundances in protein and transcript during PTI in this study (Figures 4C, 5B). *Sl*POD12 was also induced in transcription after inoculation of *Fusarium oxysporum f. sp. radicis-lycopersici* (Manzo et al., 2016). These results suggest that the enzymes responsible for the ROS burst at different infection stages are different, *Sl*POD12 mainly plays a role in the early stage, while *Sl*RBOHB contributes to the sustained ROS burst in the later stage.

Several proteins involved in ROS and redox homeostasis were differentially regulated in PTI and ETI. Acting as ROS scavenger enzymes, CAT was increased during PTI and ETI, while glutathione peroxidase was induced only during ETI (Figure 5C and Supplementary Table 2). Similarly, several CAT proteins from potato (*Solanum tuberosum*) were reported to increase in ETI, and a sugarcane ScCAT2 was found to play a positive role in plant immune responses (Resjö et al., 2019). In addition, four proteins involved in the protein redox regulation, thioredoxin h (Trxh) and Trx-dependent peroxiredoxin were increased in ETI, and nucleoredoxin 2 and cystathionine beta-synthase domain-containing protein 1 (CBSX1) were increased in PTI (Supplementary Table 2). The pathogen-inducible nucleoredoxin was found to play a critical role in maintaining the reduced state of CAT (Kneeshaw et al., 2017), and the CBSX1 was involved in the activation of plastidial Trxs (Yoo et al., 2011). Several proteins associated with the reduction of oxidative damage were altered during ETI (Supplementary Table 2), including an ETI-increased glutathione S-transferase known for detoxification of xenobiotics and lipid peroxides, and two ETI-decreased germin-like proteins involved in plant immune responses (Davidson et al., 2009). The increases of these ROS and redox homeostasis related proteins during ETI/PTI, together with their functional implications from previous studies, shed new light on potential redox regulatory mechanisms (e.g., how redox cross-talks with phosphorylation) underlying the ETI/PTI interaction.

### Transcription, Protein Turnover, Transport and Trafficking Altered by Pathogen Infection

The transcriptional reprogramming of immune-related genes is regulated at multiple levels. In this study, several proteins involved in histone modification and chromatin remodeling (e.g., histone H2A, histone H1, XH/XS domain-containing protein, methyl-CpG-binding domain-containing protein 1), as well as transcriptional regulation (e.g., RNA-binding proteins, splicing factors and Zinc finger transcription factors) were decreased during ETI (Supplementary Table 2). Growing evidence indicates that innate immunity and RNA silencing are closely linked. Remarkably, transportin-1 and argonaute2a, two proteins related to RNA silencing were both induced in ETI. Arabidopsis transportin 1 is a transport receptor for two small RNA-binding proteins, AtGRP7 and AtGRP8 (glycine-rich RNA-binding protein 7 and 8), which are involved in plant immunity (Ziemienowicz et al., 2003). Argonaute 2A functions to promote antibacterial immunity by binding miR393b^∗^, which mediates silencing of a Golgi-localized SNARE (soluble N-ethyl maleimide sensitive factor attachment protein receptor) gene, Qb-SNARE Membrin12 (MEMB12), and depressing exocytosis of PR proteins (Zhang et al., 2011). Our results provide new clues for understanding how these two proteins might be involved in ETI. In this study, many ribosomal proteins were decreased in ETI, and two elongation factors were induced in PTI (Supplementary Table 3). Notably, heat shock protein (Hsp) 90-1 and Hsp70 were both ETI-induced at the transcriptional level and protein level (Figure 4C). The Hsp90 and Hsp70 family members are known to play key roles in protein folding and cellular signal regulation under various stresses. Plant Hsp90 has been well-characterized as a core component of various protein complexes that associate with co-chaperones such as tetratricopeptide repeat (TPR)-type or non-TPR-co-chaperones. For example, Hsp90 interacts with RAR1 (required for Mla12 resistance) and SGT1 (suppressor of the G_2_ allele of *skp*1) that contain a TPR domain essential for RPS2-mediated disease resistance (Takahashi et al., 2003; Park and Seo, 2015). The induced Hsp90-1 transcript and protein abundance might have a certain inherent relationship with the enhanced phosphorylation of several TPR-like proteins (Supplementary Table 3).

Many defense molecules are secreted into the extracellular space during pathogenesis. One way is secretion through ABC transporters (ABCTs) in the plasma membrane, and the other is exocytosis mediated by SNAREs. Our results revealed that two ABCTs (ABCB27, Solyc03g114950.2.1; PDR, Solyc09g091660.3.1) were increased in ETI at the transcription and protein levels, one more (Solyc11g069090.2.1) was increased at the protein level, and phosphorylation of another ABCT (Solyc09g008240.3.1) was decreased at Ser-679 in PTI (Supplementary Tables 2, 3). An ABC transporter was shown to confer broad spectrum resistance against multiple fungal pathogens (Krattinger et al., 2009). ABC transporter PENETRATION 3 (PEN3) is required for callose deposition, and activation for pathogen defense in *Arabidopsis* (Clay et al., 2009). Moreover, differential phosphorylation of *At*PEN3 was detected in response to flg22, and further analysis revealed that the phosphorylation may regulate the activity of PEN3 (Underwood and Somerville, 2017). Several proteins involved in vesicle transport were also altered at levels of protein abundance and phosphorylation during PTI and/or ETI. The increased Sec1 in transcription and protein levels (Figure 4C) may promote the SNARE complex formation during ETI, facilitating membrane fusion for vesicle trafficking (Karnik et al., 2013; Baker and Hughson, 2016). The ETI-increased clathrin heavy chain may be involved in the clathrin-mediated endocytosis, which is required for immunity mediated by PRR kinases (Mbengue et al., 2016). Several ARF-GTPase-activating proteins showed changes in protein abundance and phosphorylation levels during PTI and/or ETI (Supplementary Tables 2, 3). ARF-GTPase-activating proteins, associated with ADP-ribosylation factor GTPases, play important roles in the plant immunity through the regulation of vesicles (Wang et al., 2016). Additionally, phosphorylation of plant ubx domain-containing protein 8, vesicle-associated protein 1–4, and beta-adaptin-like protein was all increased in ETI, and phosphorylation of sorting nexin 1 was increased during both PTI and ETI, suggesting their functions in vesicle-mediated transport might be regulated by phosphorylation.

### Cell Wall Remodeling, Hormone Biosynthesis and Signaling in PTI and ETI

Phenylalanine ammonia lyase (PAL), a key enzyme of the phenylpropanoid pathway, plays important roles in defense metabolite biosynthesis and cell wall reinforcement (Dempsey et al., 2011; Miedes et al., 2014). An isoform of PAL (Solyc10g086180.2.1) was induced in ETI, and another isoform (Solyc09g007900.5.1) was increased in both ETI and PTI at transcription and protein levels (Figure 4C). In addition, tyrosine aminotransferase and arogenate dehydratase involved in phenylalanine biosynthesis were also increased in ETI (Supplementary Table 2). Moreover, a methylenetetrahydrofolate reductase that modulates methyl metabolism and lignin monomer methylation was also increased in ETI (Tang et al., 2014). In contrast, three proteins involved in wax biosynthetic process (i.e., fatty acid hydroxylase, HXXXD-type acyl-transferase, and transferase), another three proteins involved in the metabolism of pectins (i.e., pectin lyase, pectin esterase, and pectin acetylesterase) and an expansin were all decreased in ETI (Supplementary Table 2). These results suggest active remodeling of cell walls and waxes during ETI.

Several proteins involved in the biosynthesis and signaling of different phytohormones, e.g., jasmonic acid (JA), abscisic acid (ABA), ethylene and auxin, were found to be PTI- and/or ETI-responsive (Supplementary Table 2). Four proteins involved in JA biosynthesis were identified in this study, including ETI-induced 12-oxophytodienoate reductase 3, 3-ketoacyl-CoA thiolase peroxisomal-like and phospholipase Da1 (PLDa1), as well as both PTI- and ETI-induced PLDb1 (Figure 5C and Supplementary Table 2). Among these, PLDb1 was also found to be PTI- and ETI-induced at the transcriptional level (Figure 4C; Pombo et al., 2014). These results support the roles of these proteins in JA accumulation in tomato interaction with *Pst* DC3000 at specific stages during immune response (Zhao et al., 2013). ABA induces stomatal closure to limit pathogen entry (Melotto et al., 2008). ETI-induced aldehyde oxidase 1 that can oxidize abscisic aldehyde to ABA. SNF1 (sucrose non-fermenting 1) -related protein kinases that phosphorylate ABA-responsive element binding factors may play important roles in plant defense against pathogens (Finkelstein, 2013; Mine et al., 2017). Moreover, ACO1, the rate-limiting enzyme of the ethylene biosynthesis, was induced significantly during both PTI and ETI at transcription and protein levels (Figures 4C, 5C) (Barry et al., 1996). This is consistent with a previous study that ethylene was emitted from tomato leaves upon pathogen infection (Ciardi et al., 2000). A short-chain dehydrogenase/reductase, which functions in converting indole-3-butyric acid to indole-3-acetic acid, increased in PTI. This PTI-induced short-chain dehydrogenase/reductase may account for the elevated auxin levels in plants following pathogen infection (Wang and Fu, 2011). How different hormones crosstalk in PTI and/or ETI is an exciting future research direction.

### Newly Discovered Potential PTI- and ETI Markers

PTI reporters can be used to indicate the occurrence of PTI in the plant, thus successful development of PTI reporter can serve as a valuable tool in understanding *Pst* host-plant interactions. Pombo’s RNA-seq work identified NAC and SE as potential PTI-specific markers in tomato-*Pst* interaction (Pombo et al., 2014) and they were used subsequently to characterize PTI in multiple accessions of tomato (Roberts et al., 2019). In this study, these two PTI-specific genes were not identified as having an associated differentially expressed proteins. In order to determine whether NAC and SE are well-suited as PTI markers, promoter-GUS fusion constructs of these two genes were developed and assayed for the activity caused by PTI treatment. Circular blue stains were observed in *N. benthamiana*, where the *35S*:*GUS*, *NAC*:*GUS*, and *SE*:*GUS* agrobacteria were infiltrated, except for the YFP negative control. This may be due to constitutive or leaky expression of GUS, or other stressors such as mechanical damage and *Agrobacterium*-induced PTI. However, our results revealed that the expected overlapping pattern from the experimental design was not observed in the leaves treated with negative control buffer, flg22, and *P. fluorescens*, regardless of the PTI treatment time and GUS staining incubation time (**Figures 5, 6**). These results suggest that the promoters of *NAC* and *SE* are not PTI-inducible and therefore these genes might not be appropriate for using as PTI-reporters.

Based on our proteomic and phosphoproteomic data, as well as transcriptomic data, some proteins were identified as potential markers for PTI or ETI (Figure 1B and Supplementary Tables 2, 3, 9). For example, five and 24 proteins were specifically PTI- and ETI-induced, respectively, at both the transcript and protein levels. Some of highly changed transcripts/proteins are newly discovered potential PTI- and ETI-specific markers (Table 1 and Figure 4). With protein level evidence, these markers are more likely to reflect the PTI and ETI processes in plant cells. Additionally, six conserved phosphorylate sites were found to be responsive to PTI and/or ETI in tomato and Arabidopsis (Table 2). More hypothesis-testing experiments such as the “overlapping circle” pattern assay and molecular genetics are needed to further characterize these newly discovered potential PTI or ETI markers.

## Conclusion

Integrative analyses of the proteome and phosphoproteome in the tomato-*Pst* pathosystem using different *Pst* DC3000 mutants allowed identification of proteins and phosphoproteins potentially involved in PTI and/or ETI responses. Our proteomic results indicate that signal transduction, ROS and redox homeostasis and defense, transcription machinery and protein turnover, cell wall remodeling, as well as hormone biosynthesis and signaling play important roles in tomato against *Pst* infection. Our proteomic results, however, did not find differential levels of NAC and SE, two PTI-specific markers identified from the previous transcriptomic work. The “overlapping circle” pattern assay revealed that these two genes were not PTI-inducible using Agrobacterium transient expression in *N. benthamiana*. The ETI- and PTI-specific sets of the DR proteins and phosphoproteins revealed from this study provide a unique resource for further dissection of plant immune responses. Further functional analyses of these DR proteins and phosphorylation modifications (e.g., the specific markers) using molecular genetics and other approaches will promote advancements in the field of plant defense.

## Data Availability Statement

The proteomics data presented in this study are deposited in ProteomeXchange via PRIDE repository with the accession number PXD028240.

## Author Contributions

SC and GM conceived and designed the study. JY, JG, ZD, QS, BT, and JK analyzed the data. JG, JY, ZD, TZ, NZ, and CD performed the experiments. JY and JG wrote the draft with suggestions from coauthors. JY, GM, and SC finalized the manuscript. All authors have read and approved the final manuscript.

## Conflict of Interest

CD was employed by the Thermo Fisher Scientific Inc. The remaining authors declare that the research was conducted in the absence of any commercial or financial relationships that could be construed as a potential conflict of interest.

## Publisher’s Note

All claims expressed in this article are solely those of the authors and do not necessarily represent those of their affiliated organizations, or those of the publisher, the editors and the reviewers. Any product that may be evaluated in this article, or claim that may be made by its manufacturer, is not guaranteed or endorsed by the publisher.

## Footnotes

1
http://swissmodel.expasy.org/

2
http://www.ebi.ac.uk/interpro/

3
https://www.ncbi.nlm.nih.gov/tools/primer-blast/
