# Supplementary figures and images for: Integrative Proteomic and Phosphoproteomic Analyses of Pattern- and Effector-Triggered Immunity in Tomato
Source: Front Plant Sci. 2021 Dec 3;12:768693. doi: 10.3389/fpls.2021.768693 (PMC8677958; doi:10.3389/fpls.2021.768693)

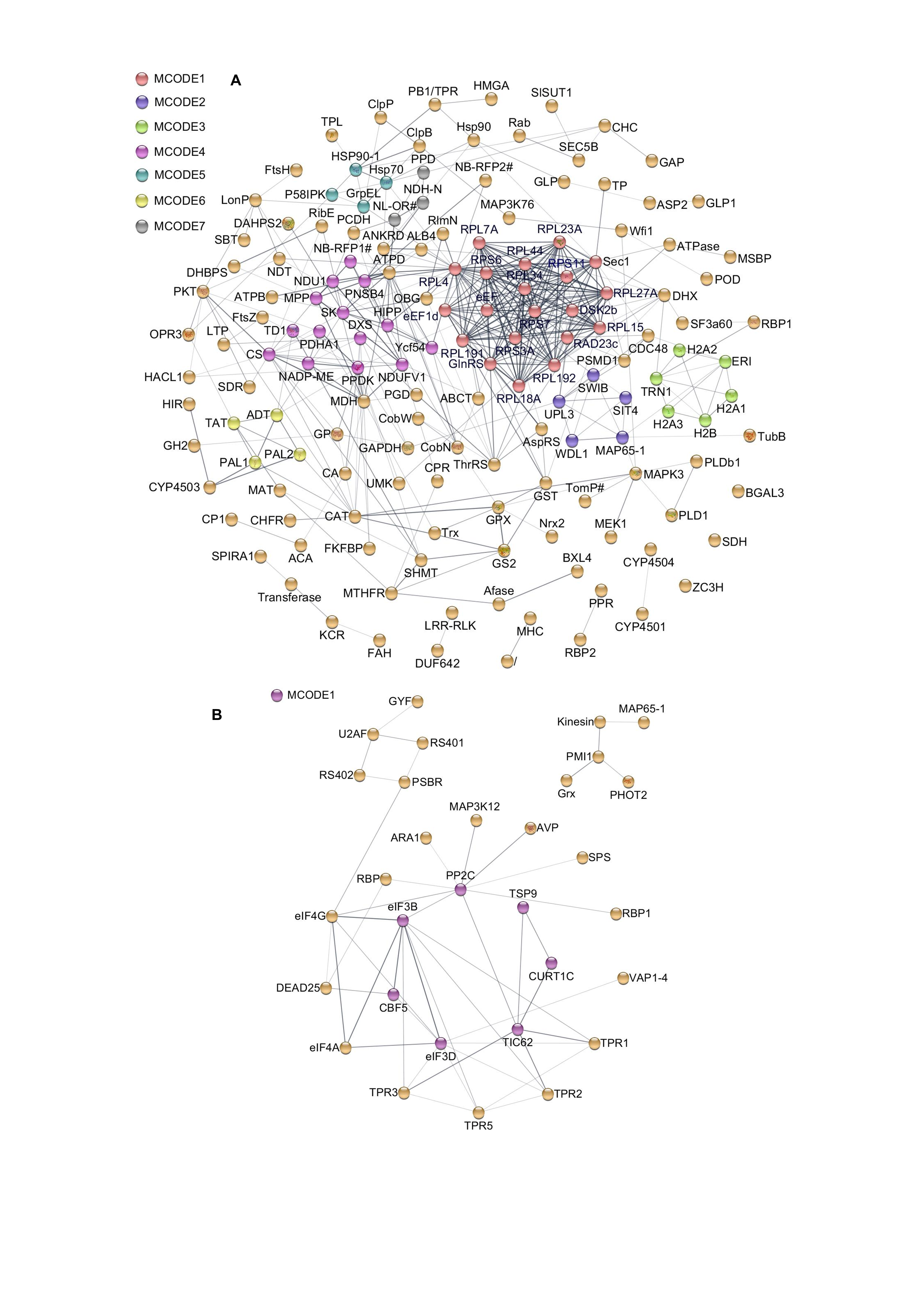

Supplement: Supplementary file 1 [file Image_1.TIF]

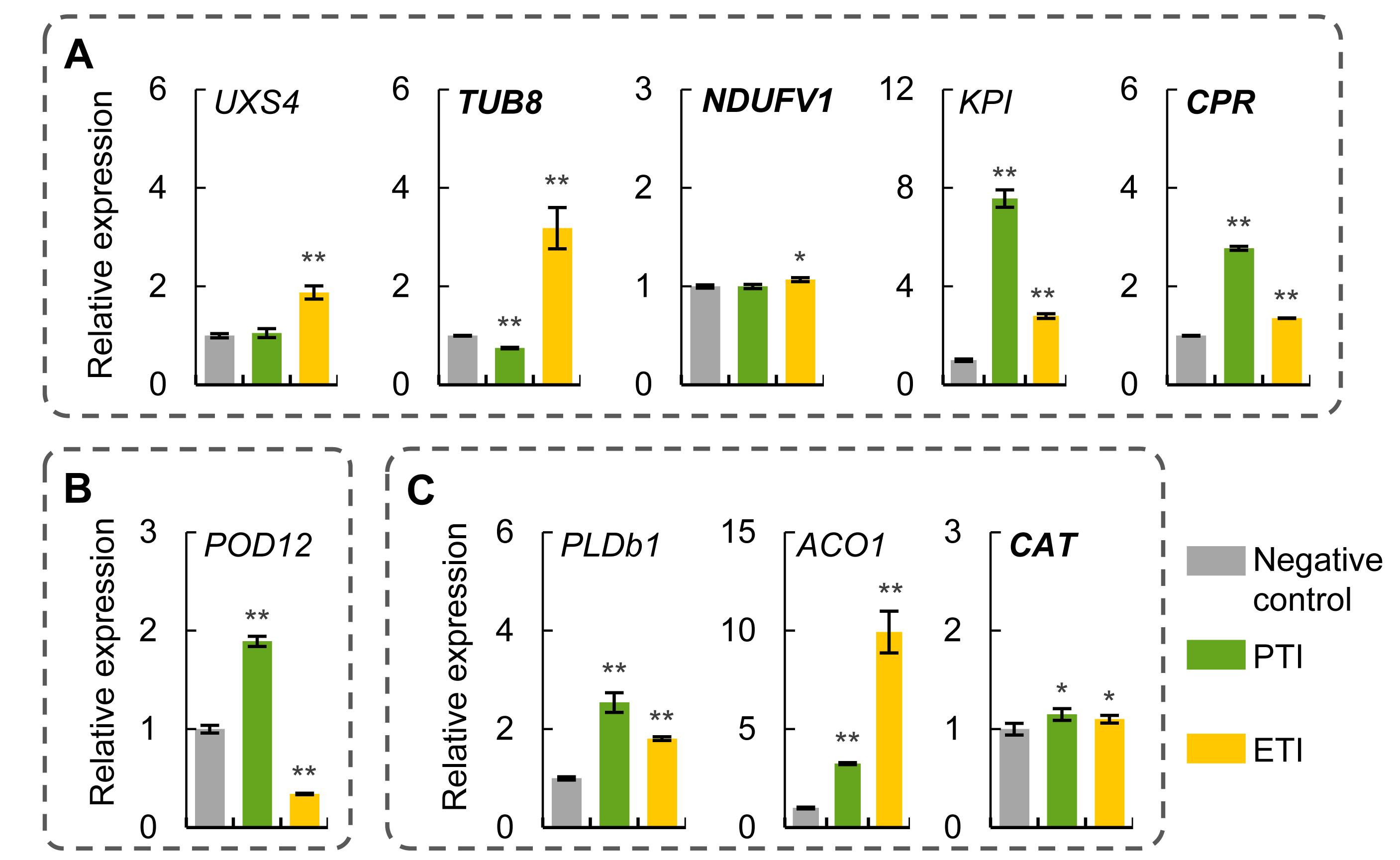

Supplement: Supplementary file 2 [file Image_2.TIF]

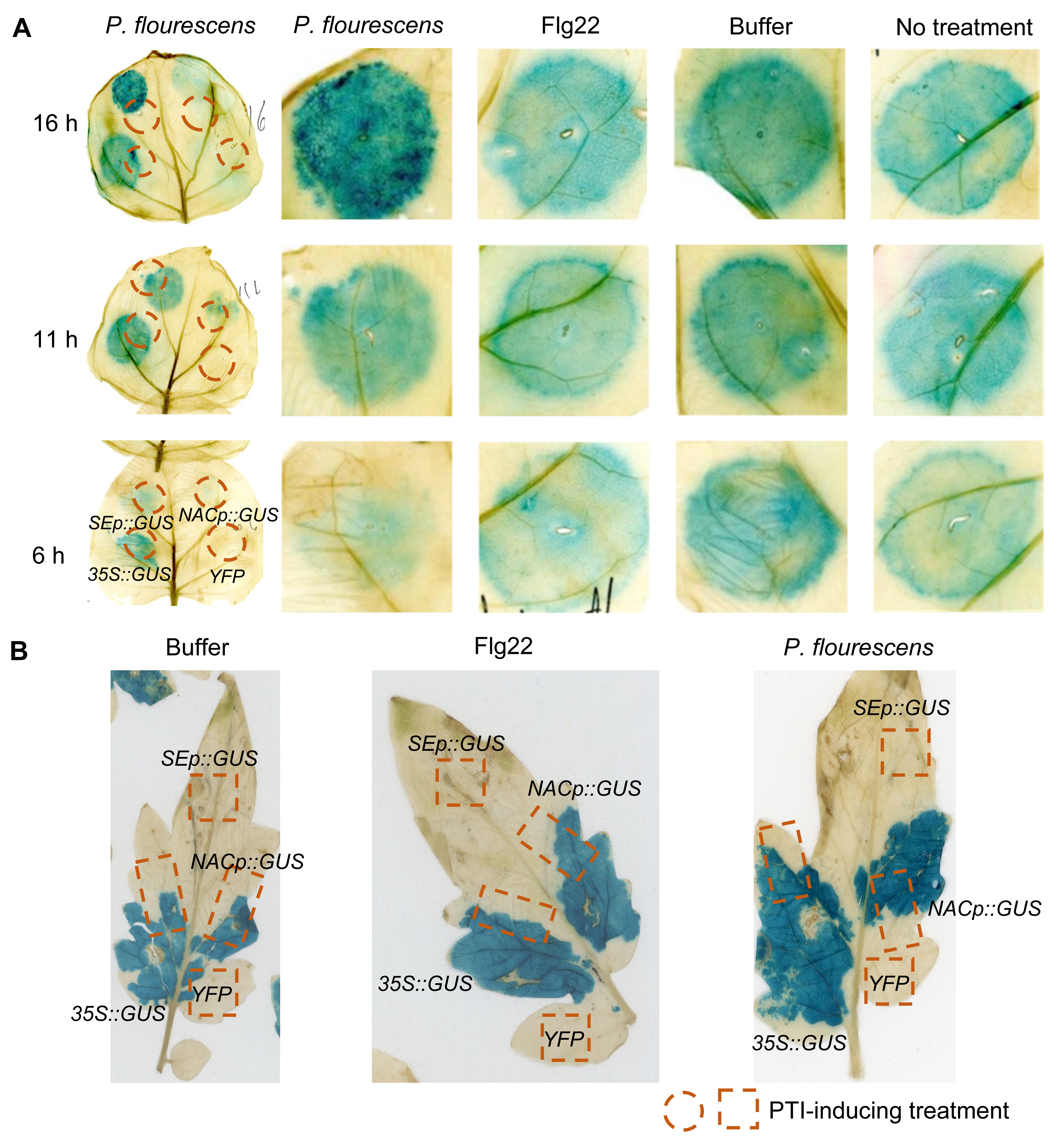

Supplement: Supplementary file 3 [file Image_3.TIF]
